# Application of Micro-Electro-Mechanical Sensors Contactless NDT of Concrete Structures

**DOI:** 10.3390/s150409078

**Published:** 2015-04-17

**Authors:** Suyun Ham, John S. Popovics

**Affiliations:** Department of Civil and Environmental Engineering, The University of Illinois at Urbana-Champaign, 205 N. Mathews Ave, MC-250, Urbana, IL 61801, USA; E-Mail: coast98@gmail.com

**Keywords:** air-coupled, impact-echo, MEMS, ultrasound

## Abstract

The utility of micro-electro-mechanical sensors (MEMS) for application in air-coupled (contactless or noncontact) sensing to concrete nondestructive testing (NDT) is studied in this paper. The fundamental operation and characteristics of MEMS are first described. Then application of MEMS sensors toward established concrete test methods, including vibration resonance, impact-echo, ultrasonic surface wave, and multi-channel analysis of surface waves (MASW), is demonstrated. In each test application, the performance of MEMS is compared with conventional contactless and contact sensing technology. Favorable performance of the MEMS sensors demonstrates the potential of the technology for applied contactless NDT efforts. Objective: To illustrate the utility of air-coupled MEMS sensors for concrete NDT, as compared with conventional sensor technology.

## 1. Introduction

High speed structural damage detection and/or monitoring of existing concrete infrastructure elements is becoming important for quality assurance and their management [1,2,3]. Mechanical wave methods, such as ultrasonic wave pulse propagation, impact-echo, and seismic multi-channel analysis of surface waves (MASW), do show sensitivity to internal damage and can be applied to concrete structures in the field. However, as normally applied, these nondestructive testing (NDT) methods utilize point-test configurations that require physical contact with the concrete structure [4]. Consequently, the coupling process is both time and labor intensive, and thus may result in undesirably slow inspection and give rise to other problems [5]. When the concrete surface is rough, surface preparation (e.g., grinding) is needed prior to testing, and in extreme cases tests simply cannot be applied. Furthermore, the sensor coupling conditions may affect received signals and disrupt measurements.

Recently, contactless mechanical wave sensors are finding increased application in NDT. The motion of a solid surface that results from mechanical wave propagation or vibration generates acoustic waves that leak into the surrounding air. Contactless sensors detect the leaked acoustic waves, from which the surface motion of the solid that results from wave propagation is inferred. Recent developments with specially modified microphones show promise for enhanced sensitivity to specific phenomena, for example microphone inside of parabolic reflectors show enhanced sensitivity to vibrations associated with the impact echo test [6]. However, conventional microphones and other existing contactless sensors may show limitations across the broad range of sonic and ultrasonic test configurations, for example poor sensitivity in terms of acoustic pressure amplitude (low signal-to-noise ratio), or frequency response (limited frequency bandwidth), large size, and external power requirements [7]. Micro-electro-mechanical sensor (MEMS) microphones represent new acoustic technology that overcome some of the existing sensor limitations, and have gained wide acceptance in many applications [8].

**Figure 1:**
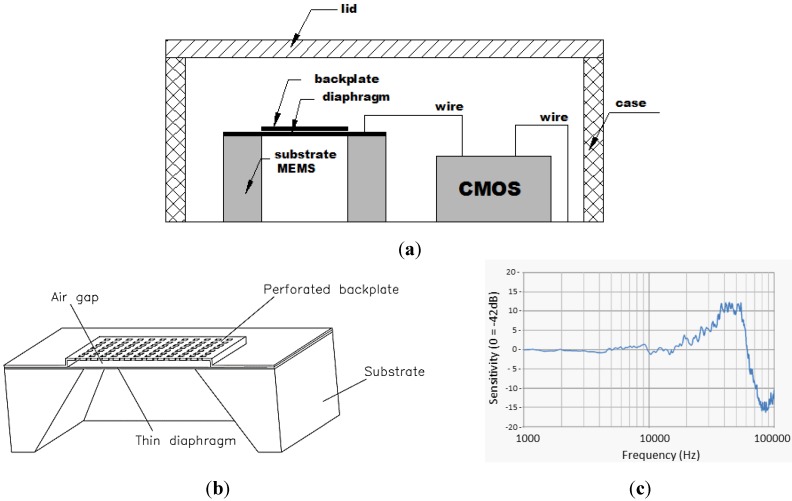
Basic construction (**a**), construction of active elements (**b**) (Reproduced with permission from [10]) and frequency response (**c**) (Reproduced with permission from [11]), of the type of micro-electro-mechanical sensors (MEMS) sensor used in this work: nominal sensitivity = 7.9 mV/Pa.

Figure 1a shows the basic construction of a MEMS device. MEMS sensors vary in package type, output format and sensitivity, where the sensitivity is principally controlled by the elastic and geometric properties of the active elements [9]. For the MEMS acoustic sensors used in this work, the active elements are composed of a fixed perforated backplate, support-body (substrate), and a thin pressure sensitive diaphragm, as shown in Figure 1b. These elements are composed principally of monocrystalline silicon and produced using micro machining thin-film technology. The operation of capacitive MEMS requires an external DC bias voltage applied between the diaphragm and back plate. This typical type of sensor is called a capacitive complementary metal-oxide-semiconductor (CMOS) MEMS. The CMOS unit provides the low bias voltage to the diaphragm. The applied bias causes the diaphragm to move towards and to come in contact with the extended edges of the back plate by electrostatic attraction. Once the diaphragm contacts the extended edges of the back plate, the boundary conditions of the diaphragm are well approximated by a simply supported plate [10]. This configuration provides an air gap between the diaphragm and back plate, and ensures an effective acoustic seal across the diaphragm. Air pressure variations, for example those caused by propagating acoustic waves in the air that impinge on the diaphragm, cause forced mechanical vibration of the stretched diaphragm. The CMOS unit converts the mechanical vibration to an electrical voltage output signal that is proportional to a specific sound field quantity, e.g., air pressure variation. The sensitivity and frequency bandwidth of the response are controlled by shape, thickness, and boundary conditions of the diaphragm. Figure 1c shows the frequency response of the capacitive MEMS sensors used in this work. The frequency response is fairly flat between 10 and 30 kHz, with increased sensitivity between 30 and 55 kHz. The sensitivity decreases significantly above 100 kHz (response not shown).

This capacitive active element design provides high sensitivity to acoustic pressure changes at high signal to noise ratio but with relatively low power consumption and small size and mass [12]. Also, the sensors are produced at lower cost than their bulk device counterparts. All of these features enable increased system design flexibility, and allow multiple MEMS components to be deployed in serial or parallel arrays to increase functionality, device capability, and reliability. These benefits offer potential for MEMS to be used to improve current NDT capability for the concrete infrastructure.

This paper introduces MEMS sensors through application to a range of existing NDT techniques: resonance vibration, impact echo, seismic MASW and ultrasonic surface wave analysis. The utility of MEMS in these applications is demonstrated by direct comparison to existing sensing technology. As far as the authors are aware, the effective application of air-coupled sensors, and in particular MEMS sensors, across the breadth of concrete non-destructive testing methods has not been reported before.

## 2. Background

### 2.1. Air-Coupled Acoustic Sensors

**Figure 2:**
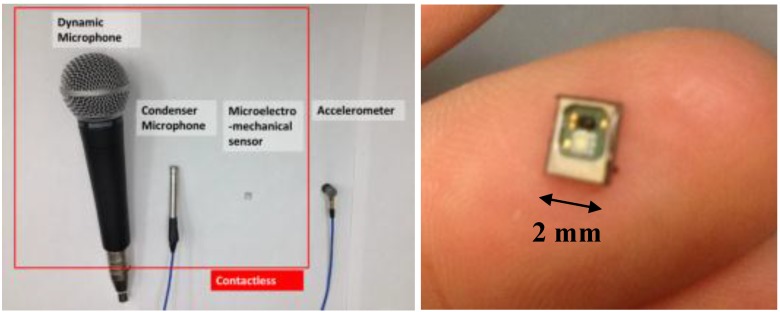
Set of tested sensors (**left**) and individual MEMS sensor unit (**right**).

In this paper, the performance of contactless MEMS sensors are evaluated with respect to those from two different contactless sensors and a contact sensor in terms of application to conventional concrete NDT methods. We present the results from an accelerometer to show relative performance of contactless sensors to established surface contact sensing technology. The two other contactless sensors are conventional microphones having distinct underlying technological bases: an electret condenser microphone (ECM) and a dynamic membrane microphone (DMM). An accelerometer is used as a contact sensor and in some cases also as reference. All sensors employed are shown in Figure 2. Conventional microphones typically exhibit reasonably high detection sensitivity, about 1.8 mV/Pa and 4 mV/Pa at 1 kHz for DMM and ECE microphones, respectively [13]. However, the working frequency range depends on the type of microphone used; conventional DMM are limited to frequencies well below 20 kHz, while ECM can sense, in some cases, up to 80 kHz [13]. MEMS sensors show typical sensitivity of 7.9 mV/Pa at 1 kHz [11] and frequency range of 1 to 90 kHz (see typical range of frequency response in Figure 1). MEMS, ECM and accelerometers require external power and pre-amplification or circuitry, while the DMM do not. Table 1 compares the performance metrics of the four different sensors employed in this work. The signal to noise ratio (SNR) is calculated as the ratio of variance of total signal to variance of noise in the signal in dB; the reported SNR values are computed from experimental data for impact-echo tests, which will be discussed subsequently. Among the air-coupled sensors, MEMS demonstrate the highest sensitivity and SNR. Furthermore MEMS sensors compare favorably to the contact accelerometer; the SNR for MEMS is about half of that for conventional contact accelerometers for the tests reported here.

**Table 1:** Performance metrics of four sensors employed in this work.

	Sensitivity (mV/Pa) at 1 kHz	Frequency Range (kHz)	SNR (dB)
Accelerometer (contact)	1.02 mV/(m/s^2^)	1 to 50 kHz	25.12
MEMS	7.9 (mV/Pa)	1 to 90 kHz	14.7
ECM	4 (mV/Pa)	4 to 80 kHz	7.04
DMM	1.85 (mV/Pa)	below 20 kHz	5.02

### 2.2. Vibration

Vibration resonance tests are employed to monitor elastic constitutive properties and accrued damage in concrete samples, following a standardized test procedure [14]. In the procedure, the vibration of fundamental resonance modes is monitored, and the associated frequency of vibration measured with contact and contactless sensors. Dynamic elastic constants of the bulk samples are calculated from the frequency values. The specific mode of vibration, among the longitudinal, flexural and torsional families of modes, is set up by the physical testing configuration, and the frequency values of the fundamental modes are normally extracted from frequency domain signals.

### 2.3. Impact Echo

The impact-echo test is an applied vibration method. A light mechanical impact event is applied to the surface of the test object, which sets up local mechanical vibration resonances in the region nearby the impact point, and resulting frequencies of vibration are monitored. The frequencies of vibration are interpreted to infer object thickness, material properties, or presence of internal defects [15]. The method is usually applied to plate-like structures because straight-forward interpretation of results is possible for that geometry. In particular, a solid (defect free) plate exhibits a single relatively high resonant frequency, while a plate that contains near-surface delamination defects exhibits multiple lower frequency resonant modes [16].

### 2.4. Ultrasonic Surface Wave

Ultrasonic surface waves, also known as Rayleigh waves in the case of very large solids, can be used to characterize near-surface properties of solid materials. The group velocity and attenuation properties of these waves reveal elastic properties and damage content of the solid material on which they propagate [17]. However, surface waves show limited penetration depth below the surface; the waves have meaningful interaction with the solid material only to about a depth of one wavelength, λ. Wavelength is related to the wave frequency by

(1) $$\text{λ}=C_{R}/\text{f}$$

where $C_{R}$ is the phase velocity of the Rayleigh wave and f is the frequency. These waves are suitable for surface region characterization of materials, for example to monitor the mechanical stiffness of the surface layers for example to indicate regions of damage.

### 2.5. MASW

A deeper analysis of material properties and geometrical conditions is enabled by multiple surface wave measurements collected several spatially distinct surface sensing locations. The spectral analysis of surface waves (SASW) method is based on two surface wave measurements, and can provide estimates of Young’s Modulus (E) and thickness of near-surface layers in an inhomogeneous layered system [18]. More recently, the multichannel analysis of surface waves (MASW) method was introduced to improve the capabilities offered by SASW. MASW employs numerous surface receivers or senders along an evenly spaced linear array. Dynamic response signals obtained from surface sensors at different offsets u(x, t) are transformed to the frequency–phase-velocity domain [19] using

(2) $$S(\omega ,\;C_{T})=\int^{\text{​}}e^{-i(\frac{\omega }{C_{T}})x\;}U(x,\omega )\;dx$$

where *U(x, ω)* is the normalized complex spectrum obtained from the Fourier transform of u (x, t) , ω is the angular frequency, $C_{T}$ is the testing-phase velocity, and *S (ω,* $C_{T}$*)* is the slant-stack amplitude for each ω and $C_{T}$. Calculating *S (ω,* $C_{T}$*)* over the frequency and phase-velocity range of interest generates a phase-velocity spectrum that is analyzed to provide estimates of elastic constants and thickness of layers in an inhomogeneous layered system [18,19].

## 3. Experimental Details

### 3.1. MEMS Modification

**Figure 3:**
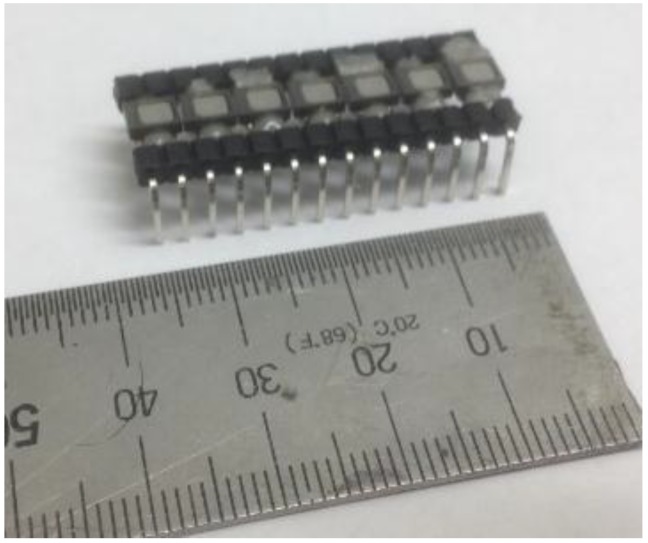
Detail of 7-element MEMS sensor array with 2-mm spacing: each MEMS unit was soldered and then coated with epoxy.

The MEMS used in this work are a commercial product. Each individual sensor unit, shown in Figure 2, requires external electrical power (1.5 to 3 direct current (DC) volts) and additional circuitry to operate; it is possible to purchase self-contained units where the sensor and required circuitry are already placed within a case. In the work reported here, however, basic MEMS sensor units were purchased, powered and wired by the authors in order to provide design flexibility needed to create multi-sensor arrays. For each MEMS sensor unit, two 0.1-μF coupling capacitors were placed at the output to form a high pass filter with input resistance, and all grounds connected to the data acquisition system (DAQ) ground. The first coupling capacitor is connected between the positive (+) and the negative (–) signal out of the MEMS sensor unit. The second capacitor is placed between the positive side DAQ and the positive side of signal out. All sensors and circuitry are electrically connected and fixed by manual soldering using a micro tip, and later encased with an epoxy coating to protect the connections from physical and mechanical events. Multiple MEMS units may be incorporated into a single array set, as shown in Figure 3. Each sensor unit requires modest power, less than 250 μA of current at potentials between1.5 to 3.6 V. In the work reported here, a single 1.5 V dry-cell battery (AAA or AA type) is used to provide the power for all the sensors in an array. The MEMS sensors provided consistent performance and sensitivity with the described power configuration.

### 3.2. Vibration Tests

**Figure 4:**
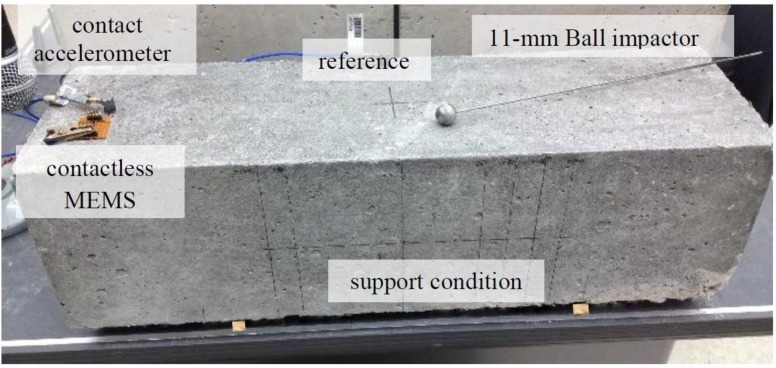
Vibration test configuration showing contactless MEMS sensor and contact accelerometer. Sample is 150 × 150 × 510 mm concrete prism.

Standard resonance vibration tests were carried out on a 150 × 150 × 510-mm mature concrete prism concrete sample with a water to cement ratio of 0.38 and a maximum aggregate size of 2 cm. Figure 4 shows the resonance testing configuration on the simply supported sample. The resonance vibrations were set up by an impact event of an 11-mm diameter steel-ball, and the resulting vibration motion was detected by both a contact accelerometer and a single MEMS sensor. We present the results of the MEMS sensor to show relative performance to a standard, contact-sensing method with respect to the completely contactless MEMS sensor. One contact accelerometer was attached near the end of the simply supported beam and used to collect vibration data to compare with that collected by the nearby MEMS sensor. A second accelerometer was attached at the center of the beam to provide a timing trigger for the data collection process. The height (air gap) of the MEMS sensor above the sample was also varied to study the effects of sensor placement. The contact accelerometer was connected to a power source and preamplifier, and the signal was transferred to DAQ. The data were sampled using 16-bit resolution at a sampling frequency of 1 MHz. Each time signal had a duration of 2 ms, which provides frequency domain resolution of 500 Hz. The contactless MEMS sensor was directly connected to the same DAQ. The signal acquisition process was carried using the LabVIEW Signal Express program. The digitized time data were then processed using a Fast Fourier Transform algorithm to convert power/frequency response using MATLAB.

### 3.3. Impact Echo

**Figure 5:**
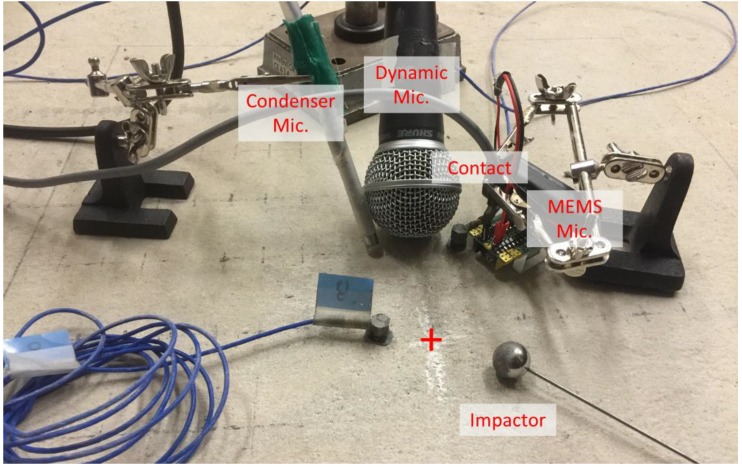
Impact echo test configuration showing three air-coupled sensors and contact sensor above the delamination region.

Impact echo tests were carried out on a mature steel-reinforced concrete slab cast from a single batch of concrete with a maximum aggregate size of 2 cm. The 28-day compressive strength of the concrete is 42.3 MPa. The slab is nominally 250-mm thick with 1500 by 2000-mm lateral dimensions, and contains a simulated 400 × 600-mm shallow delamination defect placed at a depth, 60 mm below the surface. The defect is simulated with a double layer of thin polymer sheeting placed before the concrete was cast. The P-wave velocity of the mature concrete, determined by ultrasonic pulse velocity measurement, was 4100 to 4200 m/s. Figure 5 shows the testing configuration with three different types of contactless (air-coupled) sensors and a contact accelerometer on top of the concrete slab. The transient point loading is applied through an impact event from an 11-mm steel ball applied on the surface of the concrete. The distances between the impactor and each receiving sensor are identical, and a 2-cm air gap above the concrete surface was maintained for all contactless sensors. An additional contact accelerometer was attached near the impact site in order to provide a timing trigger for the data collection process. The data were acquired using 16-bit resolution at a sampling frequency of 1 MHz. Each time signal had a duration of 2 ms, which provides frequency domain resolution of 500 Hz. Impact echo data were obtained from two different regions: at a defect-free (solid) area and above a shallow delamination.

### 3.4. Ultrasonic Surface Wave

**Figure 6:**
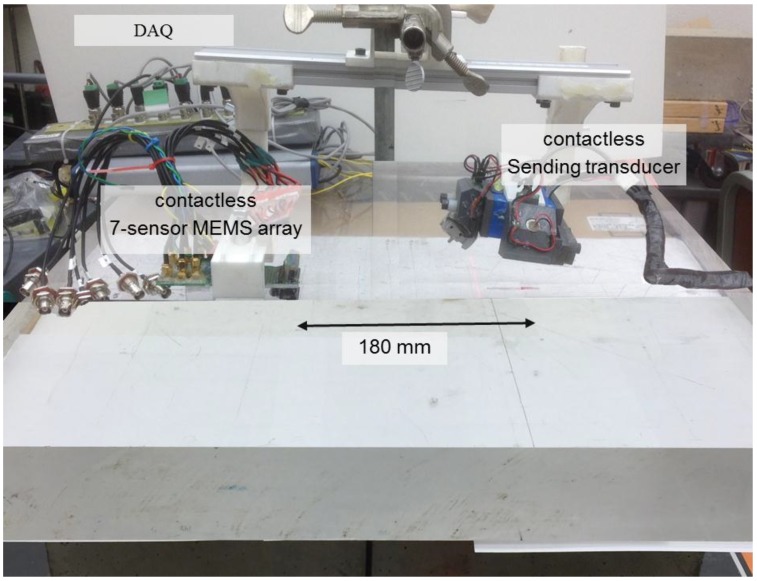
Ultrasonic surface wave testing configuration on the Poly-methyl methacrylate (PMMA) sample; 7-sensor MEMS array used to receive waves.

Airborne (fully contactless) ultrasonic surface wave tests were carried out on samples of homogeneous polymer, Poly-methyl methacrylate (PMMA), and concrete. Figure 6 shows the testing configuration on. An electrostatic-type transducer launches 50 kHz 16-cycle tone burst ultrasonic pulses in to the air toward the solid surface at an incident angle equal to the third critical angle (approximately 80° in PMMA and concrete) in order to maximize the amount of acoustic wave energy in the air transferred to propagating surface wave energy in the solid. A 7-element MEMS transducer array is used to detect the propagating surface wave signals, which leak acoustic waves in to air as they propagate. The height (air gap) of the sending transducer was 52 mm above the surface and that of the MEMS array was 5 mm. The data from the array were acquired using a 16-bit resolution at a sampling frequency of 2 MHz. Each time signal had a duration of 0.2 ms, which provides frequency domain resolution of 500 Hz.

Two different tests were carried out. The first test set evaluates the consistency and accuracy of the contactless test set up through tests carried out on a 1 × 1 × 0.15-m PMMA slab sample. The PMMA sample was used for these tests because of its uniform and well established wave propagation properties. Different tests were carried out such that the axis of the 7-sensor MEMS-array was configured to be perpendicular (2-mm spacing) and parallel (10-mm spacing) to the direction of the propagating surface waves. The emitting transducer and MEMS array were separated by 180 mm in the first test as shown in Figure 6.

The second test evaluates the effectiveness of the contactless test set up for concrete evaluation through tests carried out on a 300 × 300 × 600-mm mature concrete beam sample. A 7-sensor MEMS array with 10 mm spacing between sensor units was used to detect the signals. The axis of the array was configured to be parallel to the direction of surface wave propagation. After collecting a set of signal at one position, the array was moved 70 mm along a line away from the first position and another set of data was collected. Therefore, 14 spatially distinct signals were collected. Data were also collected using a contact accelerometer at the same 14 sensed locations, where the accelerometer was placed at points every 10 mm from 80 to 210 mm along a line away from the fixed sending position. The pulse arrival time was automatically determined for each signal using a statistics-based criterion, which calculated a reasonable threshold value based on the character of the noise region before the surface wave arrival within each time signal [20].

### 3.5. Seismic MASW

**Figure 7:**
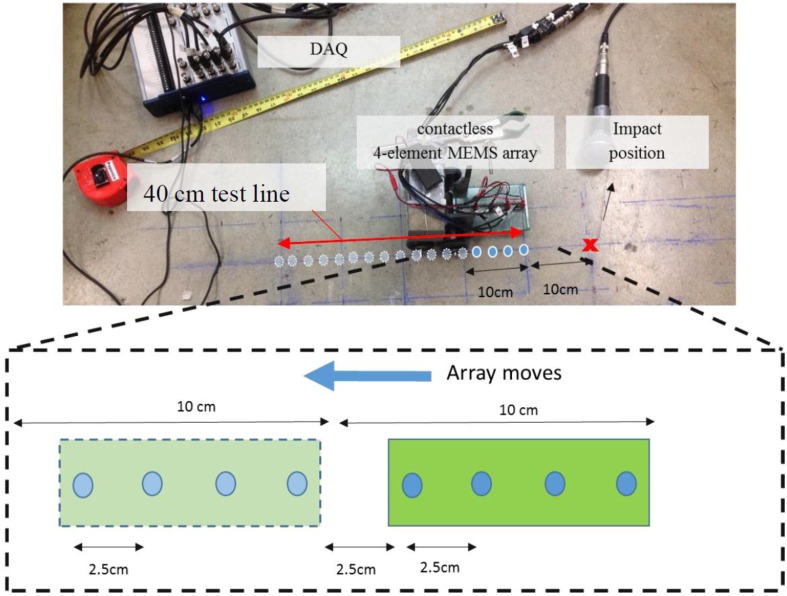
Seismic MASW test configuration showing air coupled sensors. Sensed positions indicated by blue circles.

Seismic MASW tests were carried out on a mature concrete floor slab. The thickness of the floor slab is 200 mm. The data were collected from a contact accelerometer and a 4-element MEMS sensor array that has 2.5-cm spacing between sensors. The test configuration is shown in Figure 7. The seismic waves were generated by an impact event from a 10-mm diameter steel-ball applied at one surface point with the sensors at a fixed position. The sensors were then moved along a line away from the impact point by a fixed amount, and another impact event applied and seismic data set collected. This procedure was repeated to give a total of 16 signals across a test line 40 cm in length. The four-element MEMS sensor array set was moved a total of four times, while the single contact sensor was moved a total of sixteen times at a spacing of 2.5 cm. The data were acquired using 16-bit resolution at a sampling frequency of 1 MHz. Each time signal had a duration of 2 ms. The time signal data were used to generate phase velocity dispersion curves following the MASW algorithm [21] described in Section 2.5.

## 4. Results and Discussion

### 4.1. Vibration Tests

**Figure 8:**
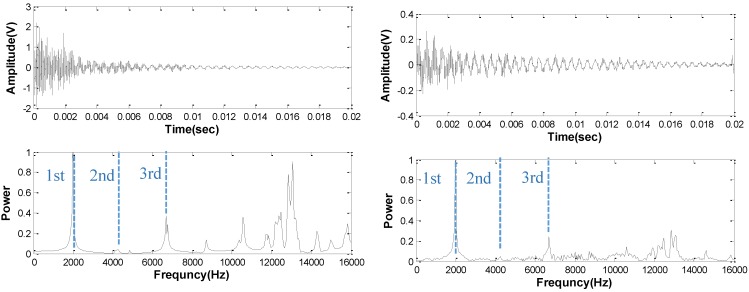
Time (**top set**) and normalized frequency power (**bottom set**) signals obtained from vibration tests on concrete prism. Signals for a MEMS sensor (**right set**) compared to those from a contact accelerometer (**left set**). Expected vibrational mode frequencies indicated by dashed lines (1.96, 4.08 and 6.5 kHz).

Figure 8 shows the response of the concrete beam to the transverse mode resonance vibration tests, where data from a contact accelerometer (left) and MEMS (right) are shown. The expected frequencies of the first three flexural modes, as determined by a Finite element (FE) Eigenmode analysis carried out by the authors, are 1960, 4080, and 6502 Hz, respectively. As expected, the MEMS sensor shows lower overall signal amplitude and poorer signal to noise ratio compared the contact sensor. However, both spectra clearly indicate a fundamental response at 1950 Hz and another higher response at 6505 Hz. These responses match very well with the expected first and third vibrational modes. Neither sensor detects the second mode well, likely because of the sample support, sensor position, and excitation configuration used. The MEMS sensor provides the same results as the contact accelerometer up to the first three modes. Higher frequency responses are seen in the accelerometer response, however, which the MEMS do not show as clearly.

**Figure 9:**
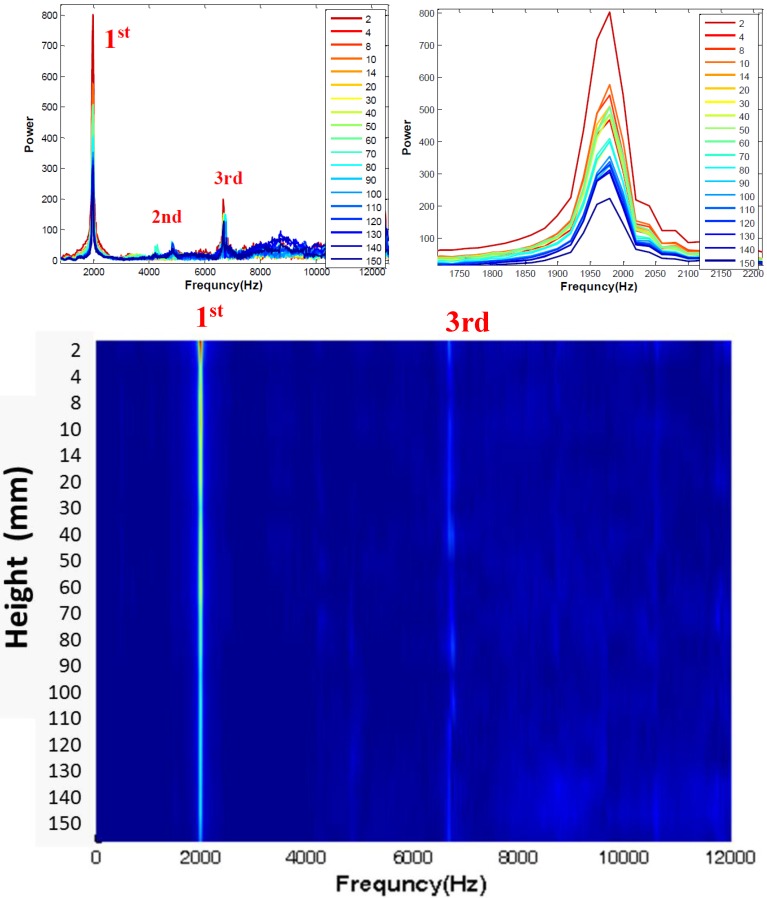
Study of effects of MEMS sensor height. Tests carried out above a 150 × 150 × 510-mm concrete prism. Frequency signals (**top**); full signal (**top left**) and first portion (**top right**) obtained with sensor heights ranging from 2 to 150 mm. Stacked frequency plot of collected data (**bottom**); x-axis indicates frequency and y-axis indicates height. Spectral power indicated by image brightness.

Now, the effect of MEMS sensor height (air gap distance) is considered. Figure 9 shows the spectra from 19 vibration tests with varying MEMS height from 2 to 150 mm above the concrete surface. Conventional spectra are shown in the top left and right of the figure, while a stacked b-scan presentation of the same data is shown at the bottom. The results demonstrate that the MEMS sensor provides consistent and clear results at all sensed heights, and that the frequency values are not affected by height. However, the signal amplitude (power) decreases with sensor height, and it appears that beyond 150 mm, the signal data are more difficult to discern, especially for the higher order mode.

The authors also carried out vibrational tests on 100 × 200-mm concrete cylinders (results not shown here) and good performance of MEMS sensors was confirmed.

### 4.2. Impact Echo

**Figure 10:**
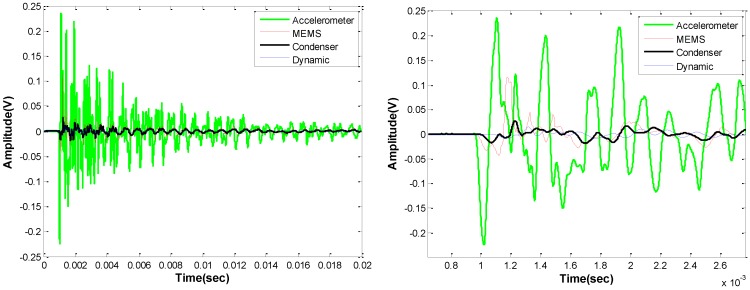
Impact echo time data collected above a 400 × 600 mm rectangular shallow delamination using different sensors; full signal (**left**) and first portion of signal (**right**).

**Figure 11:**
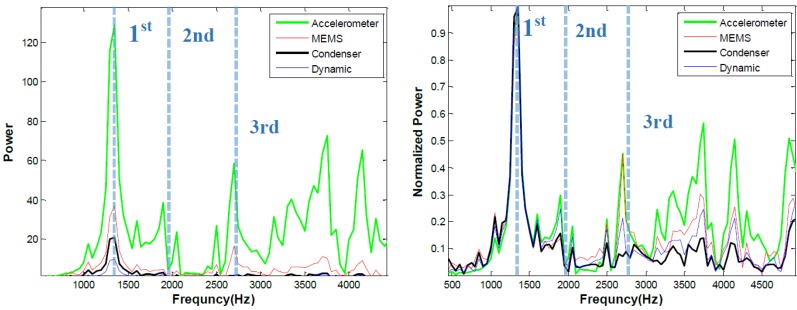
Impact echo frequency data collected above a 400 × 600 rectangular shallow delamination using different sensors; absolute data (**left**) and normalized data **(right**). Frequency of first three expected vibrational modes indicated with dashed lines.

**Figure 12:**
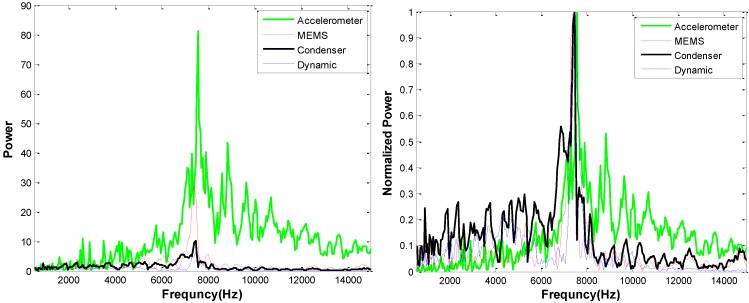
Impact echo frequency data collected above a solid 250 mm thick slab using different sensors; absolute data (**left**) and normalized data (**right**).

Impact echo results collected using several different sensors, are shown in Figure 10, Figure 11 and Figure 12. Figure 10 shows time signals from the contact accelerometer, and contactless MEMS, ECM, and dynamic membrane microphone (DMM) sensors. As seen in Figure 10, the time signals show a broad range of amplitude and phase. As expected the contact accelerometer exhibits the highest signal amplitude. Among the contactless sensors, MEMS exhibits the highest amplitude and SNR, as shown in Table 1. The maximum amplitude in the accelerometer, MEMS, ECM, and DMM time signals are 0.23, 0.115, 0.026, and 0.014 V, respectively. The conventional air-coupled sensors show much lower responses than the MEMS do in these tests. These findings are also seen in the associated frequency spectra shown in Figure 11. Multiple frequency resonance modes are expected at 1375, 2250, and 2950 Hz in this case; the frequencies of the first three expected modes are indicated, using a procedure described in [16], in the figure. The second mode is not seen in the impact echo responses, likely because of relative sensor and excitation point positions over the delamination defect. All sensors clearly indicate the first expected resonant mode at 1.33 kHz regardless of spectral amplitude, as seen in the normalized spectral plots in Figure 11 (right). However, only the accelerometer and the MEMS clearly indicate the third mode, likely because of limited frequency bandwidth response and low sensitivity of the DMM and ECM sensors.

Data from impact-echo tests carried out over a solid region of the slab (no underlying defects) are shown in Figure 12. The expected impact-echo mode is 7.8 kHz for this 250 mm thick slab. The spectral amplitudes of signals from the solid slab are lower than those collected over the delamination. But again, the accelerometer and MEMS responses show significantly higher amplitude than the ECM and DMM sensors. All four sensors do indicate the expected mode at the correct frequency, although the responses from the ECM and DMM sensors are obscured by poor signal to noise ratio.

### 4.3. Ultrasonic Surface Wave

**Figure 13:**
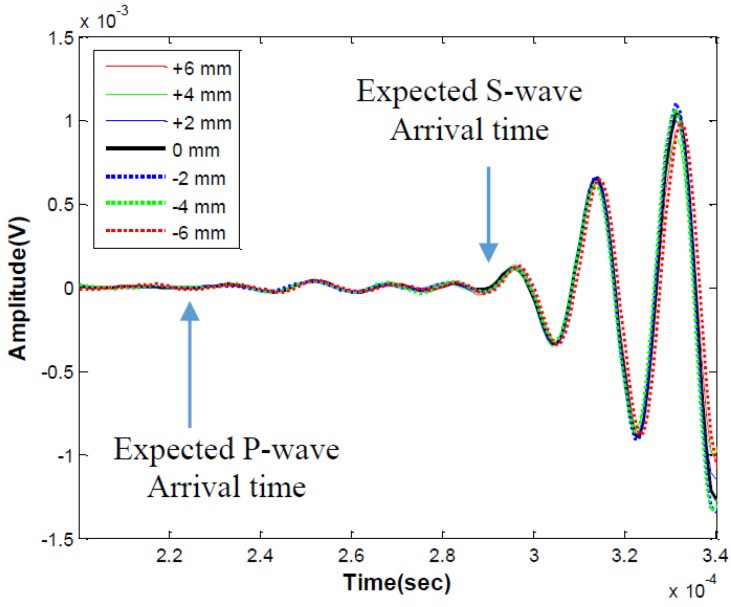
Time signals obtained from MEMS array perpendicular to surface wave propagation on PMMA; seven sensors are spaced 2 mm apart in the array.

**Figure 14:**
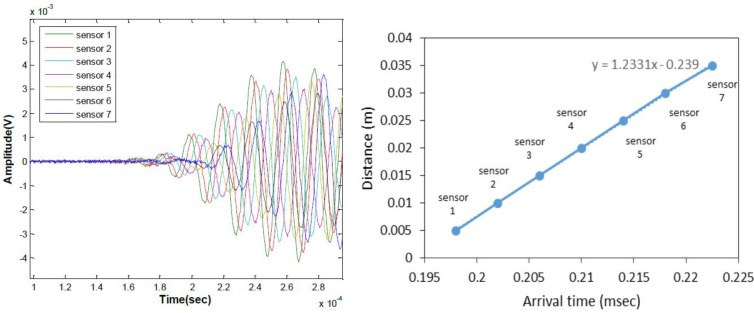
Ultrasonic surface wave signals collected with sensor array along wave path on PMMA; time signals (**left**) and presentation surface wave arrival time (**right**).

Figure 13 and Figure 14 show the ultrasonic surface wave results collected with MEMS from the PMMA sample. Preliminary tests were carried out to verify contactless sensor consistency in terms of amplitude and time of flight, where the seven-sensor MEMS array is oriented perpendicular to the direction of surface wave propagation. The sensor array has 2 mm spacing between the sensor elements, and the center sensor element, indicated by 0 mm offset in Figure 13, was aligned with the approximate center of the propagating wave beam field. Although there are slight offset distances between the sensor elements, the distances between the ultrasonic sending transducer and all seven receiving sensors are approximately 180 mm. Thus similar time signals, in terms of amplitude and phase, are expected from all sensors. The results in Figure 13 confirm that the contactless MEMS sensors provide very consistent performance across the sensor array, showing the clear arrival of the surface wave pulse slightly after the expected S-wave arrival time for PMMA. These results also indicate the potential of contactless MEMS sensors for reliable wave amplitude (attenuation) measurements that are free from sensor coupling and surface condition variations that are expected with contact sensors.

Ultrasonic surface wave velocity can be measured when the axis of the MEMS array is oriented to be parallel to the direction of wave propagation. In this case, we expect the arrival time of each seven sensors to consistently increase with increasing sensor offset distance. The results are shown in Figure 14 (left). Clear pulse arrivals with increasing delay time are seen. The pulse arrival times are plotted against sensor offset distance in Figure 14 (right), showing a strong linear relation between the two. The slope of a line fit to the data indicates a pulse group velocity of 1233 m/s, which agrees very well with the expected surface wave speed for PMMA. The results demonstrate that the air-coupled test configuration reliably and consistently monitors propagating surface wave characteristics from a known solid sample.

**Figure 15:**
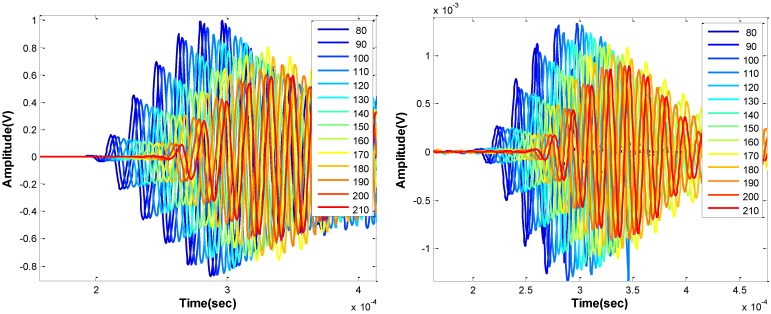
Ultrasonic surface wave time signals collected with sensor aligned along wave path on concrete; contact accelerometer (**left**) and with noncontact MEMS (**right**).

**Figure 16:**
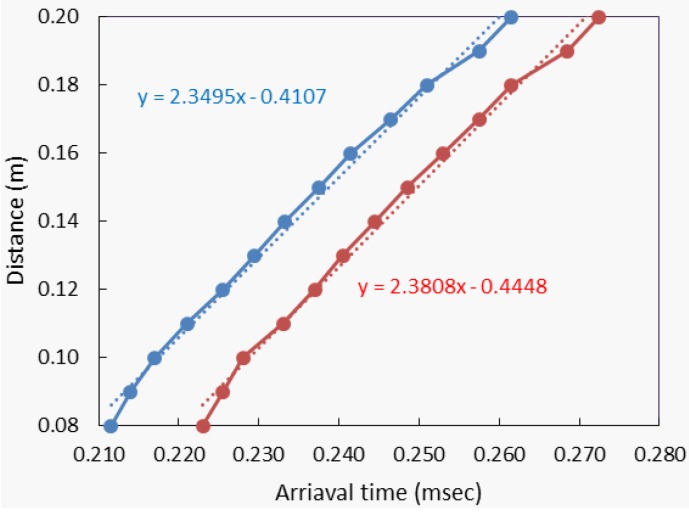
Presentation of surface wave arrival times derived from data shown Figure 15; contact accelerometers shown with blue and with contactless MEMS with red. The slope of the fit lines indicates surface wave group velocity.

Figure 15 shows results from the ultrasonic surface wave test configuration applied to concrete using the MEMS sensor array and a contact accelerometer. Figure 15 (left) shows time domain signals from the contact sensors and (right) from contactless MEMS. Both sets of signals show similar shape, although the amplitudes from the contact sensors are much larger, as expected. Figure 16 plots the determined pulse arrival time against the sensor offset distance using the data shown Figure 15. Again, the arrival time and sensor offset show a strong linear relation, although the data sets show a consistent lateral offset, owing to the air gap of the contactless MEMS sensors above concrete surface. Regardless of the offset, the slopes of the fit lines should indicate surface wave group velocity. The dataset for the contact sensors, indicated by blue points, shows a surface wave velocity of approximately 2350 m/s, while that from the MEMS sensors shows approximately 2380 m/s. Although the data show more variation than that obtained from PMMA, the slope values show good agreement with each other and are reasonable values for mature concrete. The results suggest that reliable and accurate surface wave velocity data are obtained using the fully contactless ultrasonic configuration.

### 4.4. MASW

**Figure 17:**
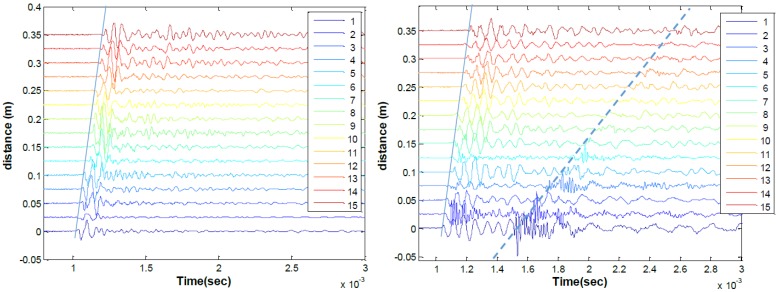
Seismic MASW time signals from sensor array collected from 200 mm thick concrete slab; data from contact accelerometers (**left**) and MEMS (**right**). Blue solid lines indicate expected surface wave arrival and dashed line indicates expected acoustic wave arrival.

Seismic time signal data collected from a 15-sensor set are shown in Figure 17, for both contact accelerometers and contactless MEMS. Both the contact (left) and contactless (right) sensor data indicate the clear arrival of a surface guided wave, as indicated by the solid line. The approximate group velocity of that pulse is 2300 m/s for both data sets. The MEMS data set, seen in Figure 17 (right), also shows the arrival of the direct acoustic wave, indicated with the dashed line have having a group velocity of 343 m/s. Although the arrival of the acoustic wave disrupts the surface wave signal of interest, meaningful information about the surface wave is provided in the signal before the acoustic wave arrival.

**Figure 18:**
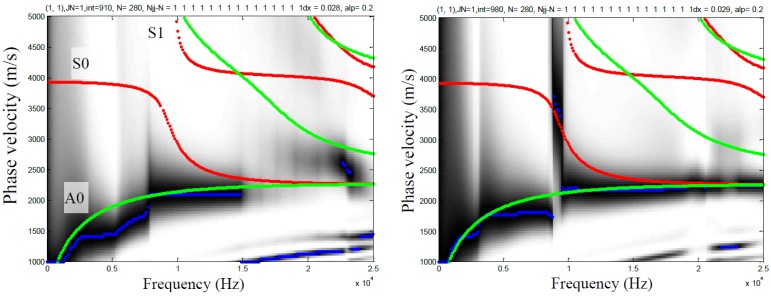
MASW dispersion curves computed from seismic time signals from 200 mm thick concrete slab; data from contact accelerometer (**left**) and MEMS sensors (**right**). Red and green lines indicate expected asymmetric and symmetric, respectively, Lamb wave modes.

Figure 18 shows the MASW dispersion curve data field calculated from the time data in Figure 17. The analytically computed Lamb wave curves are overlaid on the MASW dispersion curve field, where green lines indicate anti-systematic Lamb modes and red lines indicates symmetric Lamb modes. The Lamb wave curves are computed assuming a plate thickness of 200 mm and bulk wave velocities of the material of 4058 m/s (P-wave) and 2485 m/s (S-wave). The MASW dispersion curve fields for contact (left) and contactless (right) sensors show reasonable agreement with each other at lower values of frequency and phase velocity, although distinct difference are also seen elsewhere. In particular, the results from the MEMS show excellent agreement with the fundamental anti-symmetric (A0) mode at all displayed frequencies, while the data from the accelerometer show agreement only up to 15 kHz. The A0 mode converges to the Rayleigh surface wave at increasing frequency. The response from the MEMS sensors show a strong vertical response at approximately 9 kHz, while the response from the accelerometer does not. This response frequency coincides with the minimum frequency of the first symmetric (S1) mode, which is known to be associated with the impact-echo mode of the plate [22]. The results indicate that contactless sensor can be used to obtain meaningful MASW data, and in fact may provide more information than that provided by contact sensors.

## 5. Conclusions/Outlook

The following conclusions are drawn based on the results presented in this paper:

- MEMS sensors offer significant advantages, such as low cost, small size (good spatial resolution), broad frequency bandwidth, high SNR, and relatively easy implementation in multi-sensor arrays without excessive cost or knowhow. Multi-sensor arrays offer testing and data collection advantages that empower surface wave and MASW tests.
- Contactless MEMS sensors work well for standard vibrational resonance tests on concrete samples. The fundamental flexural mode is accurately indicated across a broad range of sensor heights.
- Impact echo flexural (delamination defect) and full thickness (solid slab) mode responses are detected by all contactless sensors and the indicated frequency values agree with those from contact accelerometers, matching expected values. MEMS show the highest sensed amplitude among contactless (air-coupled) sensors.
- MEMS sensors show very consistent ultrasonic surface wave detection in terms of time signal amplitude and phase.
- Seismic data from both contact accelerometers and contactless MEMS agree with the expected MASW analytical solution for a 200 mm plate. MEMS arrays offer accurate and reliable MASW data in a convenient testing configuration.
